# Structure-Function Relationship of SW-AT-1, a Serpin-Type Protease Inhibitor in Silkworm

**DOI:** 10.1371/journal.pone.0099013

**Published:** 2014-06-05

**Authors:** Cheng Liu, Yue Han, Xi Chen, Wei Zhang

**Affiliations:** Department of Biochemistry and Molecular Biology, College of Life Science, Nanjing Agricultural University, Nanjing, Jiangsu, China; Uppsala University, Sweden

## Abstract

Although SW-AT-1, a serpin-type trypsin inhibitor from silkworm (*Bombyx mori*), was identified in previous study, its structure-function relationship has not been studied. In this study, SW-AT-1 was cloned from the body wall of silkworm and expressed in *E. coli*. rSW-AT-1 inhibited both trypsin and chymotrypsin in a concentration-dependent manner. The association rate constant for rSW-AT-1 and trypsin is 1.31×10^−5^ M^−1^s^−1^, for rSW-AT-1 and chymotrpsin is 2.85×10^−6^ M^−1^s^−1^. Circular dichroism (CD) assay showed 33% α-helices, 16% β-sheets, 17% turns, and 31% random coils in the secondary structure of the protein. Enzymatic and CD analysis indicated that rSW-AT-1 was stable at wide pH range between 4–10, and exhibited the highest activity at weakly acidic or alkaline condition. The predicted three-dimensional structure of SW-AT-1 by PyMOL (v1.4) revealed a deductive reactive centre loop (RCL) near the C-terminus, which was extended from the body of the molecule. In addition to trypsin cleavage site in RCL, matrix-assisted laser desorption ionization time of flight mass spectrometry indicated that the chymotrypsin cleavage site of SW-AT-1 was between F336 and T337 in RCL. Directed mutagenesis indicated that both the N- and C-terminal sides of RCL have effects on the activity, and G327 and E329 played an important role in the proper folding of RCL. The physiological role of SW-AT-1 in the defense responses of silkworm were also discussed.

## Introduction

Serpins (serine-proteinase inhibitors) are a broadly distributed family of protease inhibitors, and most of the members in this family have an intrinsic inhibitory specificity against serine proteases [1]. Serpins are found in several important physiological regulation systems, such as blood coagulation and fibrinolysis in human [2], DNA binding and chromatin condensation in chicken erythrocytes [3], [4], dorsal-ventral axis formation and immuno-regulation in insects [5], [6], embryo development in nematodes [7], and the control of apoptosis [8]. Serpins have a hyper-variable amino acid (Aa) sequence around its reactive site, usually at the C-terminus. This region formed a surface loop protruding from the body of the molecule called the reactive centre loop (RCL). Besides RCL, the conserved three dimensional structure of serpins also contains 8–9 alpha-helices and 3 beta-sheets [9].

Serpins have been purified and cloned from different insects. In 1990, four serpins were isolated from the hemolymph of fifth instar larvae of *Manduca sexta*, two of which are specific to chymotrypsin, one is specific to elastase and one is to trypsin [10]. All of the *M. sexta* serpins are encoded by the same gene, and each of them is produced by alternative splicing of the final exon. *B. mori* is the lepidopteran model insect and has important economic value. Until now, Thirty-four *B. mori* serpins (BmSPI1-34) have been identified and their reactive sites were predicted [11]. Most of them could be involved in defense against bacteria and *B. bassiana*, suggesting that the silkworm serpins play regulatory roles in defense responses. Among these serpins, only four members have been studied. *Bmserpin-2* with 374 Aa residues was expressed in all developmental stages of *B. mori* larvae and various larval tissues, and located in the cytoplasm. Expression pattern analysis indicated that Bmserpin-2 may be involved in *B. mori* antiviral response [12]. Another antichymotrypsin serpin (sw-Achy) was cloned from larval fat body. The protein contains 384 Aa residues, with a preceding 16-amino-acid signal peptide. The reactive site of sw-Achy with α-chymotrypsin was identified as Thr343/Ser344 [13], [14]. In 1980’s, two serpins were identified in the hemolymph of *B. mori*. One is silkworm antichymotrypsin II (sw-AchyII), and the other is silkworm antitrypsin (SW-AT) [15], [16]. The sequence from amino terminus to residue 336 was completely identical between sw-AchyII and SW-AT. However, the degree of similarity between SW-AchyII and SW-AT from residue 337 to the carboxy terminus was only 46% [17]. The cDNA of SW-AT has an open reading frame which encodes a 392-amino acid residue polypeptide comprising a 16-residue signal peptide and a 376-residue mature protein of MW 41805 Da [16]. Similar with the serpins in *M. sexta*, four splice variants were designated as SW-AT-1, SW-AT-2, SW-AT-3, and SW-AT-4. These isoforms showed tissue-specific expression patterns, with SW-AT-1 present in almost all tissues and sw-AT-4 found in only a few tissues. Among the stress treatments, low temperature had the greatest effect on isoform expression, and the expression was also upregulated with mycotic infection [18]. Sequence alignment in our previous work found that SW-AT and SW-AchyII in the reports [15], [16] is actually SW-AT-1 and SW-AT-2 [18], respectively.

Although the SW-AT-1 was cloned and identified as a serpin family member, the study about its structure-function relationship is still lacking. In this study, we cloned SW-AT-1 from silkworm body wall and conducted heterologous expression in *E. coli*. Enzymatic and structural analysis indicated that SW-AT-1 has the inhibitory specificity both to trypsin and chymotrypsin, and the N- and C-terminal sides of its RCL are important for the activity.

## Materials and Methods

### Materials

Silkworms (Fifth instar larvae of strain P50) were purchased from the Seri-cultural Research Institute, Chinese Academy of Agricultural Sciences. Fresh larvae were quickly frozen in liquid nitrogen, and then stored at −80°C until use.

### Cloning, Expression and Purification of Recombinant His-tagged SW-AT-1 Protein

Total RNA was prepared from the silkworm body wall, and reverse-transcribed into cDNA. The full length SW-AT-1 cDNA (GenBank ID: FJ613793) was amplified using gene-specific primers 5′-CG*GGATCC*ATGGCCGTCACAAATCTCTCTAATG and 5′-CCG*CTCGAG* ATTTATAAAGATTCCGTTAAACATA (*BamH*I and *Xho*I site are shown in italics). The sequence-confirmed PCR products were gel-purified with AxyPrep DNA Gel Extraction Kit (Axygen, China) and double-digested with *Bam*HI and *Xho*I, and then cloned into the prokaryotic expression vector pET-28a. The recombinant plasmids with six histidine residue-tag were transformed into *E. coli* C43. The expression was induced by 0.4 mM isopropyl-β-D-1-thiogalactopyranoside (IPTG) at 37°C for 3 h. Cells were harvested and resuspended in lysis buffer (50 mM NaH_2_PO_4_, pH 8.0, 300 mM NaCl). After ultrasonic disruption on ice for 20 min, samples were centrifuged at 10000 g for 20 min. The resulting supernatant was collected and loaded into Ni-NTA resin column (GenScript, China). After washing the column, His-tagged SW-AT-1 was eluted with elution buffer (50 mM NaH_2_PO_4_, pH 8.0, 300 mM NaCl, and 250 mM imidazole). The purified protein was checked by 12% SDS-PAGE [19], and the protein concentration was estimated by the Bradford method with bovine serum albumin (BSA, 0.1 mg/ml) as the standard protein [20].

### The Inhibitory Activity of rSW-AT-1

The inhibitory activity of rSW-AT-1 on trypsin and chymotrypsin was determined by measuring the hydrolytic activity toward the substrates N-α-Benzoyl-D, L-arginine4-nitroanilide hydrochloride (BAPNA) and N-benzoy-L-tyrosine ethyl ester (BTEE) [21], [22], respectively. The samples were incubated with 0.4 µM trypsin at 37°C for 2 min in assay buffer (10 mM Tris–HCl, pH 8.2). After incubation, 2 mM BAPNA was added, and then incubation of another 10 min at 37°C, the reactions were stopped by adding 200 mL of 10% acetic acid. Chymotrypsin inhibitory activity was determined by incubating 0.1 µM chymotrypsin with suitable quantities of samples for 15 min at 25°C, in the presence of BTEE. The changes in absorbance was monitored at 410-nm for trypsin activity, and 256-nm for chymotrypsin activity. One trypsin or chymotrypsin unit is defined as an increase of 0.01 absorbance units per 1 ml. One inhibition unit is defined as one unit of enzyme which was inhibited.

### Stoichiometry of Inhibition

Assays for binding between rSW-AT-1 and trypsin (16 nM) or chymotrypsin (16 nM) were performed in a volume of 100 µl in 96-well microtiter plates. rSW-AT-1, its concentration ranged from 0–32 nM for trypsin and 0–40 nM for chymotrypsin, was incubated with trypsin or chymotrypsin for 30 min at 25°C. Substrate was added to a final concentration of 4 mM, and then further incubated for 10 min. The velocity of substrate hydrolysis was measured using a microplate reader. The partitioning ratio of the inhibitor-enzyme binding was determined by plotting the fractional activity (velocity of the inhibited enzyme reaction/velocity of the uninhibited enzyme reaction) versus the ratio of the initial concentrations of the inhibitor to enzyme. The X intercept was determined by linear regression analysis. As for control, trypsin and chymotrypsin were absent in the reaction mixture.

### Association Rate Constants Determination

The progressive curve method was applied to determine the interaction of SW-AT-1 with trypsin or chymotrypsin. Protease (8 nM trypsin or 8 nM chymotrypsin) was mixed with different concentrations of rSW-AT-1 and appropriate substrate (760 µM BApNA for trypsin, 250 µM BTEE for chymotrypsin). Product formation is described as below: the progressive curves were first analyzed according to *P = v_z_/k_obs_* × (1*−e* ^−*kobs*^), Where *k_obs_* is the pseudo-first-order rate constant of inhibition and *v_z_* is the initial velocity. The second-order rate constant (*k’*) was corrected for the substrate concentration, the stoichiometry of inhibition (SI) between the protease and rSW-AT-1, and the *Km* of the protease for the substrate, to calculate the *ka* as: *ka = k’* × (1+[S]*/Km*) *×*SI. All kinetic studies were repeated at least three times. Under the conditions as described, the *Km* of trypsin for BAPNA was 2.6 mM, and the *Km* of chymotrypsin for BTEE was 160 µM.

### Thermal and pH Stability

Thermal stability was tested by incubating purified rSW-AT-1 in the assay buffer for 20 min at various temperatures (37–60°C), and the samples were immediately kept on ice for 10 min. Residual inhibitory activity was measured as described above. pH stability was evaluated by measuring the residual activity after incubating purified rSW-AT-1 in different pHs (0.2 M glycine-HCl buffer for pH 2.0–4.0; 0.2 M phosphate buffer for pH6.0–8.0 and 0.2 M glycine-NaOH buffer for pH 9.0–12.0) for 20 min at room temperature. Optimal pH assay were carried out by measuring the activity at different pHs.

### Circular Dichroism

Circular dichroism (CD) measurements were carried out on an Applied Photophysics Chirascan spectropolarimeter at 25°C, equipped with a peltier-type temperature controller and a thermo-stated cell holder, interfaced with a thermostatic bath. Far-UV (185–250 nm) and near-UV (250–350 nm) spectra were recorded in 1 cm path length quartz cell at a protein concentration of 20 µg/ml in 10 mM sodium phosphate buffer. Each CD spectrum was the accumulation of four scans at 50 nm/min with 1 nm bandwidth, 0.5 s response time and 0.5 nm data pitch. CD spectra were background and buffer base corrected. The secondary structure analysis was performed using the program packages DICHROWEB and CDPro.

### Determination of Cleavage Site in SW-AT-1

To determine the reactive site at which SW-AT-1 was cleaved by chymotrypsin, the purified rSW-AT-1 (280 µg/ml) was mixed with chymotrypsin (100 µg/ml) for 10 min at room temperature, and the reaction mixture was desalted using a C_18_ zip-tip column (Millipore) and eluted with 70% acetonitrile, 0.1% formic acid. The sample was mixed with an equal volume of saturated sinapinic acid matrix on a MALDI plate, air-dried, and subjected to mass determination on a mass spectrometer (4800 Plus MALDI TOF/TOF Analyzer, Applied Biosystems, USA). The spectra were calibrated using bovine serum albumin as an external standard. The molecular mass of a peak that was absent in the control spectra of rSW-AT-1 and chymotrypsin alone was compared with calculated values of carboxyl-terminal peptides to deduce the cleavage site in SW-AT-1.

### Structural Modeling and Data Collection

Protein three-dimensional structure predictions were initiated by using SWISS MODEL (http://swissmodel.expasy.org). The program was run using the known structure of *Manduca sexta* Serpin-protease 1K complex as the template (PDB code: 1SEK, GenBank ID: AAA29334). The semi refined model was then sent to the SWISS MODEL server for final refinement. The evaluation was carried out on PSVS server (http://psvs-1_4-dev.nesg.org/), with Procheck (http://www.ebi.ac.uk/thornton-srv/software/PROCHECK/) score: 87.2% most favored regions, 12.2% additionally allowed regions, 0.6% generously allowed regions, and none disallowed regions. Images were generated in the modeling package Pymol v1.4 (http://www.pymol.org/).

### Site-directed Mutagenesis of rSW-AT-1

Site-directed mutation of particular amino acid were introduced by PCR-mediated overlap extension, and the sequences of the mutagenic PCR primers are listed in Table 1. Mutants were constructed into pET28a expression vector and expressed in *E. coli*, and the purification of mutated rSW-AT-1 were performed as described above.

**Table 1:** Primers of site-directed mutagenesis.

Code	Primer	Original amino acid	Substitute amino acid
G327A	5′-AGCAGCTTCAGCGGCTTCCTCGTTGAT-3′	Gly	Ala
A328G	5′-TGCAGCAGCTTCTCCGCCTTCCTCGTT-3′	Ala	Gly
E329G	5′-AGCTGCAGCAGCTCCAGCGCCTTCCTC-3′	Glu	Gly
A330G	5′-GTTAGCTGCAGCTCCTTCAGCGCCTTC-3′	Ala	Gly
F352A	5′-TTTGTTTGCATTGGCAACGATTGGCGG-3′	Phe	Ala
N353D	5′-CGGTTTGTTTGCGTCGAAAACGATTGG-3′	Asn	Asp
A354G	5′-AAACGGTTTGTTTCCATTGAAAACGAT-3′	Ala	Gly
N355S	5′-GTAAAACGGTTTGTCTGCATTGAAAAC-3′	Asn	Ser
K356G	5′-ATAGTAAAACGGTCCGTTTGCATTGAA-3′	Lys	Gly
P357G	5′-GGCATAGTAAAACCCTTTGTTTGCATT-3′	Pro	Gly

## Results

### Expression and Purification of Recombinant His-tagged SW-AT-1

**Figure 1:**
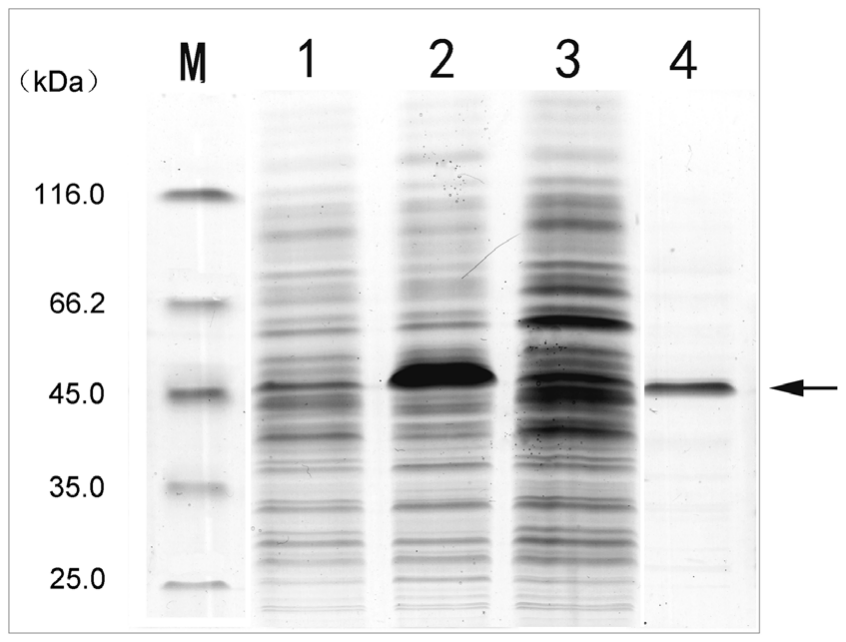
SDS-PAGE of recombinant His-tagged SW-AT-1. Lane M, molecular size markers; lane 1, pET-28a vacant vector; lane 2, total protein extracts from *E. coli* after IPTG-induction; lane 3, total protein extracts from *E. coli* without IPTG-induction; lane 4, eluted fraction after affinity chromatography. Arrowhead indicated purified rSW-AT-1.

Although SW-AT-1, also known as SW-AT in previous studies, was reported as a antitrypsin in hemolymph of *B. mori*, we identified it in body wall by mass spectrometry in our previous study (data not shown). For further biochemical characterization, we expressed recombinant SW-AT-1 (rSW-AT-1) protein in *E. coli*, and His-tagged rSW-AT-1 was purified by Ni-column (Figure 1). Recombinant His-tagged SW-AT-1 protein induced by IPTG was shown as a single 45-kDa band in SDS-PAGE, approximately corresponding to the molecular weight of 41.8 kDa [16].

### Inhibitory Activity of rSW-AT-1

**Figure 2:**
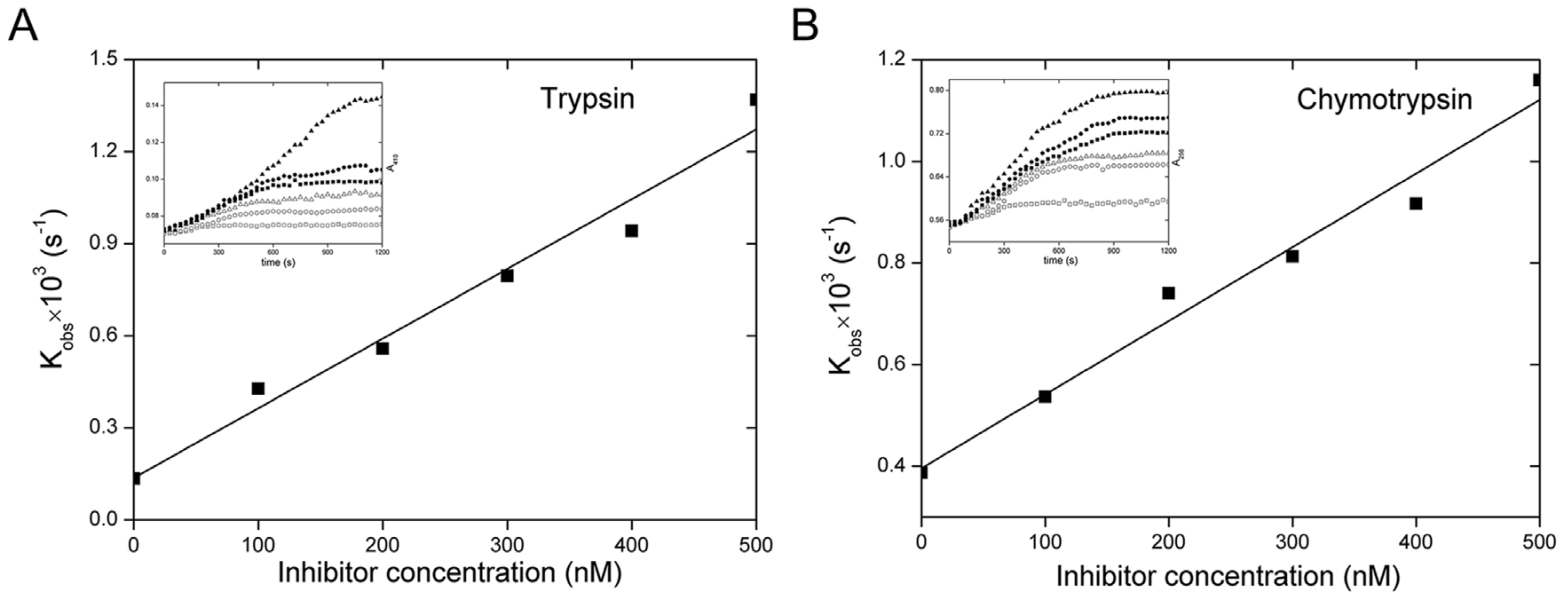
Determination of association rate constant. (A) Trypsin (8 nM) was added to a mixture of 760 µM BAPNA and SW-AT-1 at 0 (□), 100 (○), 200 (Δ), 300 (▪), 400 (•) and 500(▴) nM. (B) Chymotrypsin (8 nM) was added to a mixture of 250 µM BTEE and SW-AT-1 at 0 (□), 100 (○), 200 (Δ), 300 (▪), 400 (•) and 500(▴) nM. The progress of enzyme inactivation (*inset*) was followed by measuring absorbance at 410 nm and 256nm on a microplate reader. Pseudo-first-order rate constants of inhibition (*k_obs_*) were plotted as a function of SW-AT-1 concentration ([I]).

**Figure 3:**
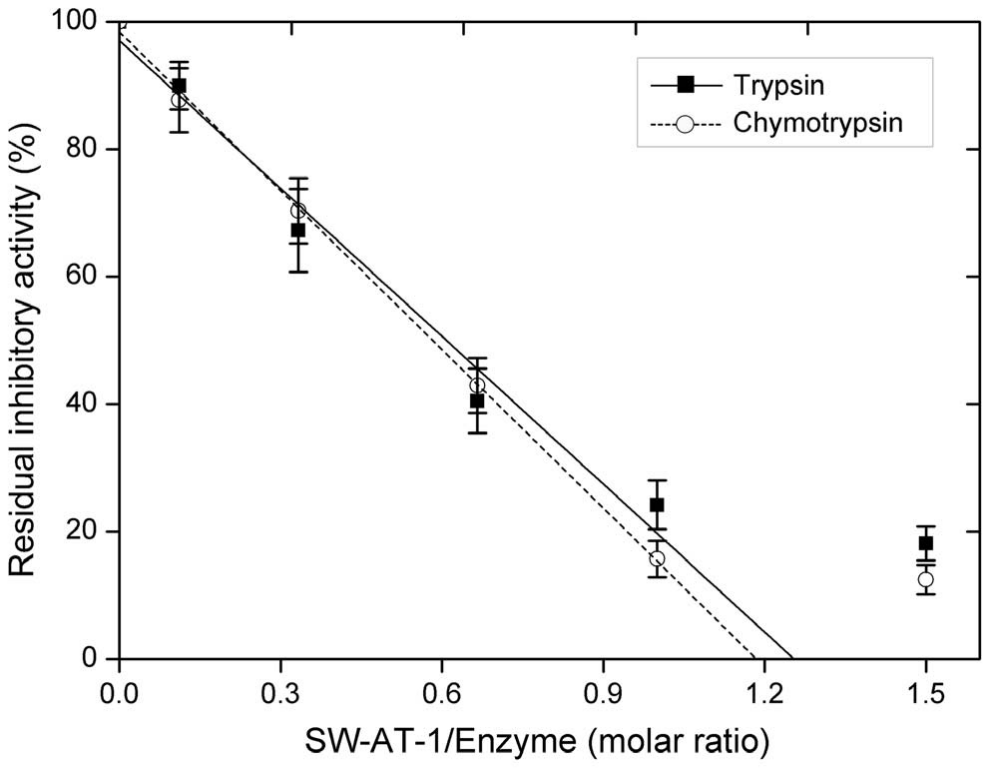
Stoichiometry of inhibition. Trypsin and chymotrypsin were incubated respectively with different concentrations of SW-AT-1 at 25°C for 20 min in the appropriate reaction buffer. Residual enzyme activity was measured by adding the appropriate substrate and determining the reaction velocity. The stoichiometry of inhibition was determined by using linear regression to extrapolate to that initial inhibitor/enzyme ratio resulting in complete inhibition of the enzyme.

It has been shown that SW-AT-1 was a trypsin inhibitor. In this study, we found that rSW-AT-1 also inhibited chymotrypsin activity. Since serpins play its inhibitory activity by forming a covalent, irreversible interaction with the protease, we determined its association rate constants (*Ka*) with trypsin and chymotrypsin. As shown in Figure 2, the second-order association rate constant (*ka*) value for the interaction between rSW-AT-1 and trypsin is 1.31×10^−5 ^M^−1^s^−1^, between rSW-AT-1 and chymotrypsin is 2.85×10^−6 ^M^−1^s^−1^. Further, rSW-AT-1 inhibited both trypsin and chymotrypsin in a concentration-dependent manner. As shown in Figure 3, when varying amounts of rSW-AT-1 were incubated with trypsin and chymotrypsin, the stoichiometry of inhibition (SI) were 1.3 and 1.2, respectively. The results indicated the high affinity of rSW-AT-1 with trypsin as well as chymotrypsin, and also suggested that rSW-AT-1 might have two reactive sites which could interact with trypsin and chymotrypsin, respectively.

### Thermal and pH Stability of rSW-AT-1

**Figure 4:**
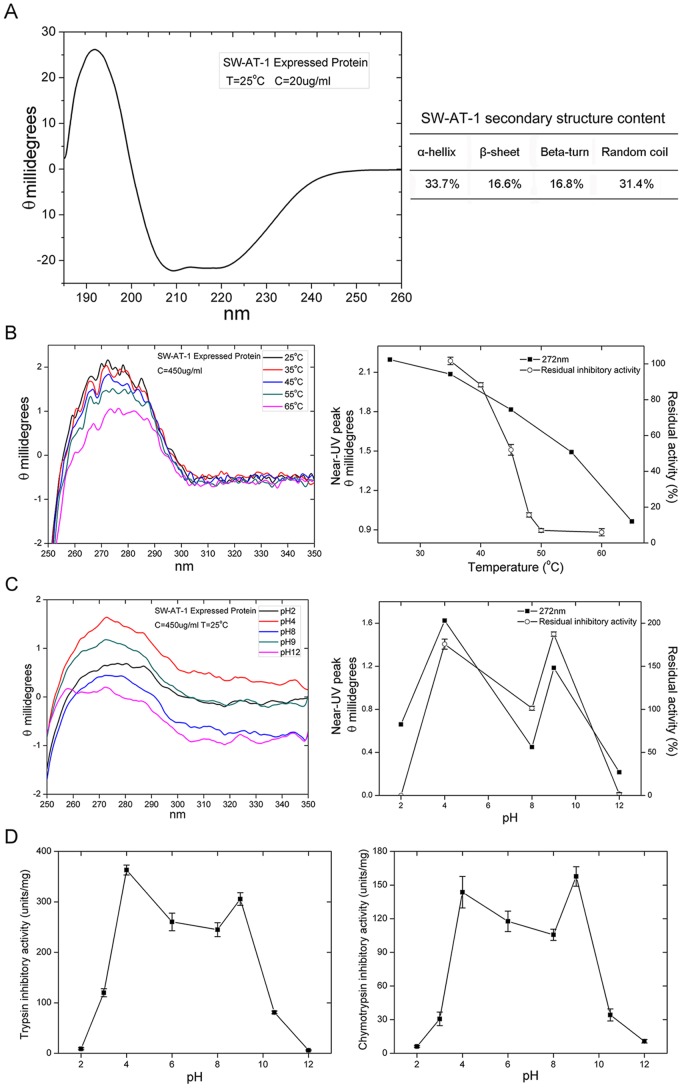
Characterization of SW-AT-1. (A) far-UV CD spectra of rSW-AT-1(left panel) and secondary sructure content (right panel); (B) near-UV CD spectra of rSW-AT-1 at different temperatures (left panel) and maximum aborbance 272-nm and trypsin inhibitory activity at different temperatures (right panel); (C) near-UV CD spectra of rSW-AT-1 at different pHs (left panel) and maximum aborbance at 272-nm and trypsin inhibitory activity at different pHs (right panel); (D) Optimal pH of rSW-AT-1 inhibitory activity for trypsin (left panel) and chymotrypsin (right panel). Bar indicates standard deviation from triplicate determination. P≤0.05 was considered statistically significant.

The secondary structure of rSW-AT-1 were analyzed by circular dichroism (CD). As shown in Figure 4A, the percentages of secondary structural elements of rSW-AT-1 showed high contents of α-helices (33.7%) and unordered structures (31.4%), and low contents of β-sheets (16.6%) and turns (16.8%). Furthermore, thermal and pH stability were monitored through near-UV CD and inhibitory activity. The tertiary structure, as shown by the 275-nm absorbance in near-UV CD, was greatly changed above 50°C (Figure 4B). Similar with the changes in tertiary structure, the inhibitory activity was reduced rapidly above 45°C, and merely lost above 50°C (Figure 4B). In contrast to its sensitivity to temperature, rSW-AT-1 exhibited stability at wide pH range between 4–10, both in tertiary structure and inhibitory activity (Figure 4C). Optimal pH assay indicated that rSW-AT-1 had the highest inhibitory activity at pH 4.0 and pH 9.0, rather than pH 7–8 (Figure 4D).

### Chymotrypsin Cleavage Site in rSW-AT-1

**Figure 5:**
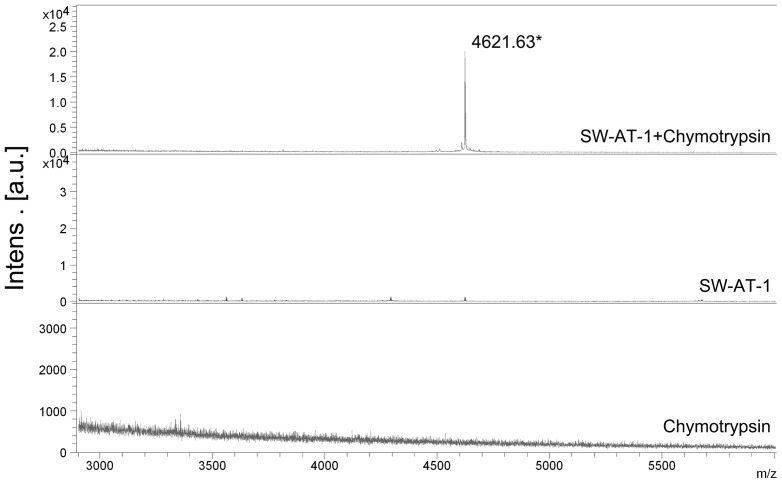
Cleavage site determination of SW-AT-1. Chymotrypsin, rSW-AT-1, and the reaction mixture of SW-AT-1+chymotrypsin were directly analyzed by MALDI-TOF mass spectrometry. A representative strong single-accumulation spectrum is presented with the mass values on top of the MH^+^ peaks at 4623.613 Da. The spectrum was subjected to noise removal and calibrated with an external standard of bovine serum albumin.

To further testify how does SW-AT-1 inhibit chymotrypsin, we used MALDI-TOF mass spectrometry to determine the reactive site at which chymotrypsin cleaves SW-AT-1. In the control sample of rSW-AT-1 or chymotrypsin alone, we did not detect any significant mass peak within the range from 3000 to 6000 Da (Figure 5). However, after incubation of chymotrypsin and rSW-AT-1, a major peak was detected at 4621.63 Da. This peak had exactly the same mass as the carboxyl-terminal peptide released from a cleavage of rSW-AT-1 between Phe336 and Thr337, which support the conclusion that rSW-AT-1 have two reactive sites which could interact with trypsin (Lys343/Val344) [15] and chymotrypsin (Phe336/Thr337), respectively.

### Both the Amino- and Carboxy-terminal Sides of Reactive Centre Loop Played Key Role in the Activity of rSW-AT-1

**Figure 6:**
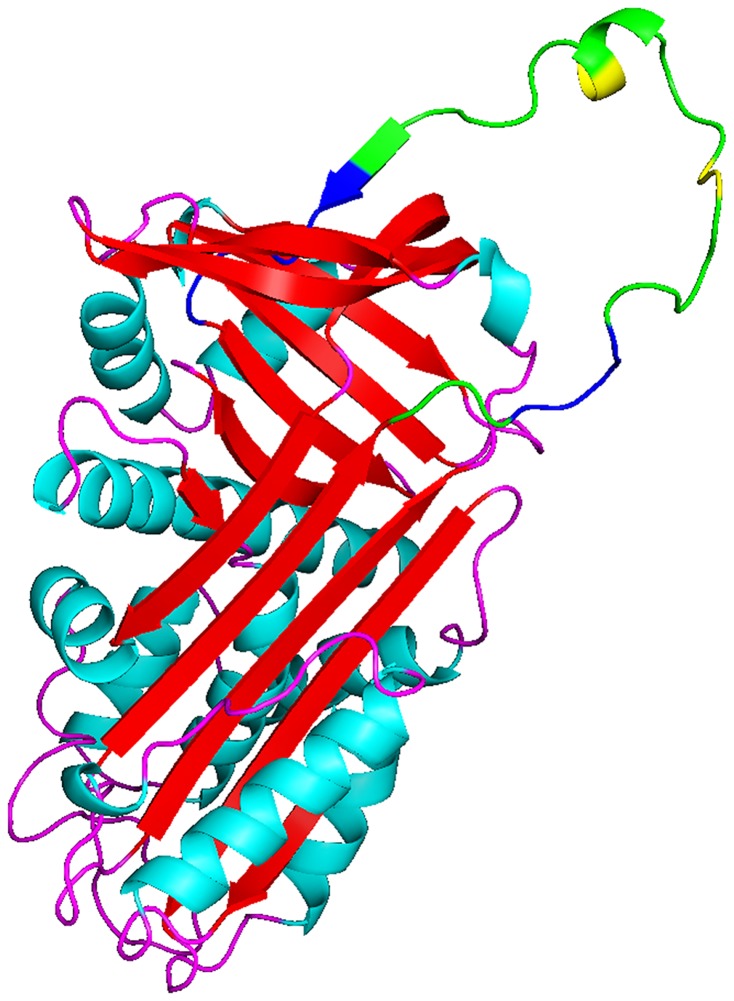
The predicted three-dimensional structure of SW-AT-1. SERPIN Domains was depicted as green, reactive site was depicted as yellow, and mutant sites were depicted as blue. The N and C termini were labeled as N and C, respectively. The structrue was generated using Open-Source PyMOL (v1.4), using *Manduca sexta* Serpin-protease 1K complex (1SEK) as the template.

In order to study the structure-funcation relationship of SW-AT-1 further, we constructed the simulated three dimentional (3D) structure of SW-AT-1, using the known structure of *Manduca sexta* Serpin-protease 1K (1SEK) complex as template. Amino acid sequence alignment showed that the identity between 1SEK and SW-AT-1 was 57% (data not shown). As shown in Figure 6, the simulated 3D structure of SW-AT-1 exhibited the common elements in the structure of serpin family members, which include 8 alpha helices, 3 beta sheets, and a extended reactive centre loop (RCL) on the top of the molecule. This putative RCL was corresponding to the variable C-terminal sequence from I323 to P357, and two reactive sites K343/V344 and F336/T337 were in the middle of the RCL (Figure 6).

**Figure 7:**
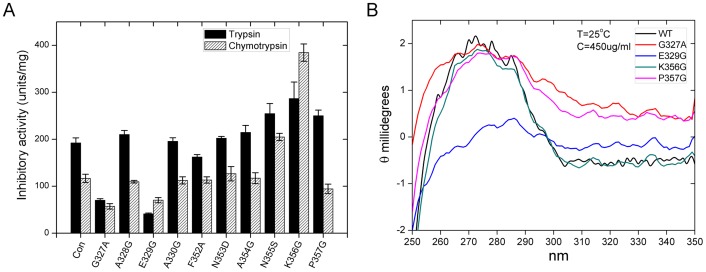
Effects of N- and C-terminal side regions of RCL on the activity of SW-AT-1. (A) Antitrypsin and antichymotrypsin activity. Data analysis was performed using the t-test function (P≤0.05). (B) near-UV CD spectra of WT and mutants.

Several studies indicated that the amino acid residues at the N-terminal side (P_19_-P_14_) of RCL are important for serpins activity [23]. In order to verify the role of the residues in N- and C-terminal side of RCL in SW-AT-1, 10 Aa residues were selected for site-directed mutation. We took K343/V344 as P_1_-P'_1_, and the selected residues were located in the P_17_-P_14_ (G327, A328, E329, A330) and P'_9_-P'_14_ (F352, N353, A354, N355, K356, P357) regions. As shown in Figure 7A, the antitrypsin activity of mutants G327A and E329G (P_17_ and P_15_) were reduced 70% to 80%, and the activity of mutants N355S, K356G and P357G (P'_12_, P'_13_, and P'_14_) were increased, compared with that of wild type. In contrast, the substitutions at A328, A330, F352, N353, and A354 sites have no effects on the inhibitory activity. With regards to antichymotrypsin activity, G327A and E329G also showed reduced level, and the mutations at K356 increased activity more than 3-folds (Figure 7A). These results demonstrated that both of the N- and C-terminal sides of RCL have effects on the inhibitory activity of SW-AT-1. CD analysis of G327A, E329G, K356G, and P357G mutants support this conclusion. As shown in Figure 7B, in consistent with its reduced activity, E329G showed significant changes in tertiary structure. In contrast, although K356G showed increased inhibitory activity both to trypsin and chymotrypsin, its structure exhibited just small changes compared with that of wild type. G327A and P357G also showed partial changes in tertiary structure.

## Discussion

Although 34 serpins have been predicted in the genome of *B. mori* [11], only few of them were studied. In the previous reports, SW-AT-1 was identified as a trypsin inhibitor [16], and the complex between SW-AT-1 and trypsin are not dissociated by SDS but are labile under high pH condition [15], [16]. However, in this study, we demonstrated that rSW-AT-1 also acts as a chymotrypsin inhibitor with a *Ka* value even smaller than that for trypsin, which indicated that SW-AT-1 has high affinity both with trypsin and chymotrypsin. This result also supported the conclusion that SW-AT-1 belongs to serpin family member because it has been well known that serpins usually have high affinity to its target proteases [24], [25]. Several trypsin inhibitors (TIs) have been demonstrated as an inhibitor with two reactive sites. For example, Bowman-Birk type was Cys-rich TIs and contained two reactive sites, which inhibited trypsin and chymotrypsin, respectively [26]. Potato-II type TIs also have two reactive sites, which inhibited both of trypsin and chymotrypsin [27], [28], [29]. In our study, we demonstrated for the first time that SW-AT-1 also have two close reactive sites in RCL, which interacted with trypsin and chymotrypsin respectively.

In this study, we analyzed the changes of SW-AT-1 structure under different temperatures and pH, although the stability and CD spectrum was roughly analyzed in previous reports [13], [14], [16], [17]. The contents of secondary elements fit quite well with the predicted 3D structure, which was mainly composed of alpha helices and a large protruding RCL (Figure 6). Although SW-AT-1 was sensitive to temperature, it exhibited stability (Figure 4C) and the highest activity (Figure 4D) under weakly acidic or alkaline conditions. Since the near-UV spectrum changed under these conditions (Figure 4C), we proposed that the shifted tertiary structure under weakly acidic or alkaline conditions may lead the reactive sites more exposure to protease, and the inhibitory activity might remain in these conditions. Since the pH of hemolymph of silkworm is between 7.6 and 8, these results also suggested that SW-AT-1 was probably not the regulator of inner proteases in silkworm, but to respond to the proteases secreted by pathogenic microorganisms [30], [31], [32]. It has been known that bacteria or fungus usually generate protease to help them to penetrate the body wall of silkworm, and this penetration will elicit a subset of tissues or hemolymph proteins to associate and form weakly acid or alkaline pH conditions [33], [34]. The highest inhibitory activity of SW-AT-1 under such conditions will prevent body wall from digestion, and ensures a localized defense reaction against the invading organisms.

Although the detailed structural changes required for inhibition of protease have yet to be worked out, it has already been clear that the serpin does undergo a major conformational change upon complex formation with protease, and the active form involves the partial insertion of the RCL into the beta-sheet of the molecule after the cleavage of RCL at reactive site [35], [36], [37], [38]. It has been reported that the regions neighboring the N-terminal side of RCL might play an important role in directing RCL as a reactive site [39], [40], [41]. In this study, G327A, E329G showed reduced inhibitory activity for both trypsin and chymotrypsin, and P357G activity for chymotrypsin was also reduced (Figure 7A). All these three mutants, especially E329G, exhibited significant changes in near-UV CD spectra (Figure 7B), which indicated that E329 is not only important for directing RCL, but also crucial for the maintenance of the whole 3D structure of SW-AT-1. Interestingly, we found that the C-terminal side of RCL also affected the inhibitory activity. Mutations at N355 and K356 with small residues such as serine or glycine promoted the inhibitory activity (Figure 7A), and the tertiary structure of K356G has only subtle changes, comparing with that of wild type protein (Figure 7B). It could be proposed that in these mutants, RCL might be expelled from beta-sheet and the P1 residue (F336 or K343) flips to an exposed protease-accessible conformation. However, such conformational rearrangement have just little effects on the whole structure of the molecule.

Taken together, we characterized SW-AT-1 as a trypsin/chymotrypsin inhibitor with two reactive sites at Phe336/Thr337 and Lys343/Val344. Both of the N- and C-terminal sides of RCL have effects on the activity, and G327 and E329 play an important role in the proper folding of RCL. Optimal pH of SW-AT-1 activity at weakly acidic and alkaline conditions enable it to play roles in defense responses in silkworm.
